# Thiol-Silylated Cellulose Nanocrystals as Selective Biodepressants in Froth Flotation

**DOI:** 10.1021/acssuschemeng.3c04013

**Published:** 2023-11-02

**Authors:** Feliciana Ludovici, Robert Hartmann, Martin Rudolph, Henrikki Liimatainen

**Affiliations:** † Fiber and Particle Engineering Research Unit, University of Oulu, P.O. Box 4300, FI-90014 Oulu, Finland; ‡ Fraunhofer Center for Chemical-Biotechnological Processes, 06237 Leuna, Germany; § Helmholtz-Zentrum-Dresden-Rossendorf, Helmholtz Institute Freiberg for Resource Technology, 09599 Freiberg, Germany

**Keywords:** aqueous silylation, nanocellulose, sulfide depressant, microflotation, sulfide ore beneficiation

## Abstract

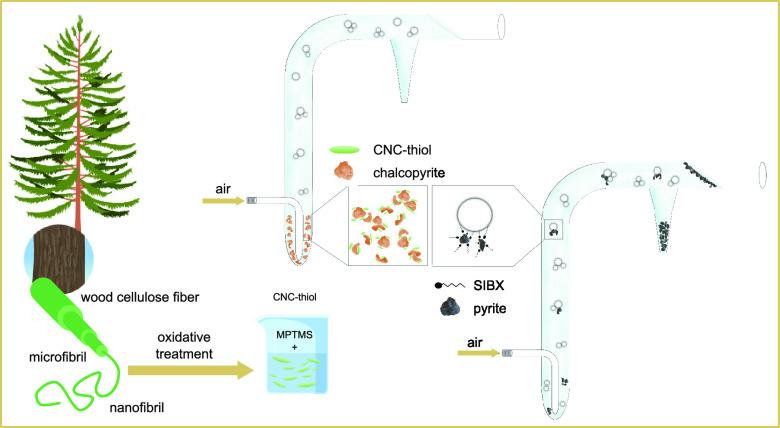

The extraction of various minerals is commonly conducted through froth flotation, which is a versatile separation method in mineral processing. In froth flotation, depressants are employed to improve the flotation selectivity by modifying the wettability of the minerals and reducing their natural or induced floatability. However, the environmental impact of many current flotation chemicals poses a challenge to the sustainability and selectivity of the ore beneficiation processes. To mitigate this issue, cellulose, particularly nanocelluloses, has been explored as a potential alternative to promote sustainable mineral processing. This study focused on silylated cellulose nanocrystals (CNCs) as biodepressants for sulfide minerals in froth flotation. CNCs containing thiol silane groups or bifunctional CNCs containing both thiol and propyl silanes were synthesized using an aqueous silylation reaction, and their performance in the flotation of chalcopyrite and pyrite was investigated in the presence of a sodium isobutyl xanthate collector. The results showed that the modified CNCs exhibited preferential interaction between chalcopyrite, and the flotation recovery of chalcopyrite decreased from ∼76% to ∼24% in the presence of thiol-grafted CNCs at pH 6, while the pyrite recovery decreased only from ∼82% to ∼75%, indicating the efficient selectivity of thiol-silylated CNCs toward chalcopyrite depression.

## Abstract 2

Silylated cellulose nanocrystals provide a new class of sustainable biodepressants for promoting the flotation selectivity of sulfide minerals.

## Introduction

Cellulose, a ubiquitous and versatile biopolymer, offers a renewable source to develop advanced, green, and eco-friendly materials and chemicals. The properties of cellulose can be manipulated through chemical modifications, but its poor solubility and degradation limit the feasibility of many treatments. Therefore, cellulose nanomaterials provide an appealing alternative source to prepare functionalized cellulose materials, as they can be directly dispersed in aqueous media without dissolution. Nanocelluloses possess high surface area, elongated structure, and numerous surface hydroxyl groups that allow one to incorporate different functional groups on the cellulose structure.^1−3^ Previously, cellulose nanomaterials have been explored in multiple applications ranging from biomedical products, electronics, and CO_2_ capture to water treatment.^4−11^ Recent studies also used nanocellulose as a mining chemical in microflotation setups and as flocculants for sulfide minerals.^12−14^

Froth flotation is a widely used separation process in mining industries for the beneficiation of complex ores. This process involves the selective attachment of minerals to air bubbles based on their wettability properties to separate the minerals from gangue using various inorganic and organic additives.^15^ Typically, surface active collectors adsorb selectively on the surface of a specific mineral, increasing its hydrophobicity and floatability. However, depressants selectively prevent the adsorption of collectors at mineral/water interfaces, rendering mineral surfaces more hydrophilic and preventing their attachment to bubbles, facilitating the selective flotation of the mineral.^16,17^

Sulfide depressants are crucial for enhancing separation selectivity in froth flotation, and inorganic and organic reagents have been broadly investigated as depressants. However, many sulfide depressants, such as sodium sulfide, sodium hydrosulfide, cyanide, and Nokes reagents, are toxic and require large quantities of chemicals, leading to high processing costs.^18−22^ Consequently, synthetic and natural organic polymers, such as polyglutamic acid (PGA), 2,3-disulfanylbutanedioic acid, lignosulfonates, thioglycolic acid, polyacrylamide, chitosan, carboxymethyl cellulose (CMC), and starch, have been explored to reduce the environmental impact of depressants.^19,20,23−25^ Although these depressants are less hazardous, they often show limited selectivity in sulfide flotation.^22,26^

The major advantage of organic depressants is their potential to be tailored through various chemical modifications by incorporating different functional groups that can effectively and selectively enhance flotations.^22^ Thus, there is considerable interest in developing and optimizing organic polymers as flotation chemicals. Cellulose, especially nanocellulose, is an appealing source of advanced, renewable, and sustainable mining chemicals. Nonetheless, the amenability of nanocelluloses toward different functionalization reactions represents a great advantage, and it can be modified with specific functional groups that render its surface wettability and enhance cellulose affinity toward specific mineral surfaces, increasing the efficiency and selectivity in mineral separation.^12−14,27,28^

Chalcopyrite is a primary copper ore resource^29^ that has a growing demand in various industries due to emerging trends of digitalization and electric transportation, such as the rise of electric vehicles. Chalcopyrite belongs to the class of sulfide minerals that generally have similar physical–chemical properties due to their semiconducting nature. However, selective separation of chalcopyrite from other sulfide minerals, such as pyrite, sphalerite, or galena, which do not bear copper in their structures, is difficult in industrial practice. Usually, sulfide minerals are well recovered in froth flotation using xanthate reagents as collectors, but the selectivity of separation is poor.^13,30−32^ Therefore, this study provides a selective separation approach in the beneficiation of chalcopyrite from sulfidic minerals, including sulfidic tailing.^33,34^

In this study, a new class of biodepressants based on cellulose nanocrystals (CNCs) that has been functionalized through an aqueous silylation reaction was introduced as a selective depressant in the froth flotation of sulfide minerals. The silylation enables efficient modification of nanocellulose substrates, and silane coupling agents presenting two reactive groups in their structure can be used for the grafting of the hydrolyzed silanols onto the cellulose with various functional groups simultaneously.^2,6,12,27,35−37^ CNCs containing thiol silane groups or bifunctional CNCs containing both thiol and propyl silane chains were synthesized to incorporate functional moieties that can both have an affinity toward chalcopyrite and modify the wettability of the cellulose.^12,27^ The grafting was performed in an aqueous reaction medium at mild temperature to promote the sustainability of the modification processes and to obtain renewable, efficient, and selective mining chemicals with controlled wettability properties.^38^

Microflotation tests were performed in an in-house built Hallimond tube, and the efficiency of the cellulose nanoparticles as depressants was investigated using sodium isobutyl xanthate (SIBX) as a collector. Specifically, the role of pH and depressant concentration in flotation recovery was elucidated. The results demonstrated that functionalized biobased nanomaterials can unlock new paths toward more sustainable mineral processing and improve the flotation selectivity of sulfide minerals.

## Experimental Section

### Materials

A commercial CNC (BGB Ultra) produced from acetate-grade dissolving pulp (Western Hemlock) using an oxidative process was purchased from Blue Goose Biorefineries (Canada) and used in functionalization reactions without any further purification.

The silylation reagents of CNC, 3-mercaptopropyltrimethoxysilane (MPTMS, 95%) and n-propyltriethoxysilane (PTS), were purchased from Sigma-Aldrich (Germany) and Gelest (USA), respectively. Ethanol (96% v/v) solution was supplied by VWR (BDH Chemicals, France). Diluted NaOH and HCl solutions (Oy FF-Chemicals Ab, Finland) were used for the pH adjustment. SIBX, which was supplied by IXOM (Australia), was used as a collector during the microflotation experiments. All reagents were used without any further purification. Deionized water (18.2 MΩcm) was used throughout the experiments.

The mineral samples, chalcopyrite and pyrite, were obtained from Mexico and Peru, respectively. The elemental composition and mineral structure of the samples have been previously published.^12^ The minerals were ground in a Retsch planetary ball mill (PM 200, Germany) by adding 50 g of minerals in each grinding jar with five stainless steel balls of 15 mm diameter. Grinding of chalcopyrite and pyrite was performed at rotational speeds of 250 and 350 min^–1^, respectively, for 3 min. Finally, with dry sieving, a coarse fraction (between 63 and 125 μm) was prepared for microflotation. For ζ potential measurements, a fine fraction size was required. Therefore, 5 g of a mineral sample was ground in a jar with 30 stainless steel balls of 6.3 mm diameter at 650 min^–1^ for 30 min. The particle size distributions were determined through aqueous mineral dispersions subjected to laser diffraction analysis (Beckman Coulter LS 13 320, USA), and the results are presented in Table S1 and Figure S1.

### Silylation of Cellulose Nanocrystals

Four different silylated cellulose nanocrystal samples, i.e., CNCs containing thiol silane groups (CNC-thiol), and mixed CNCs containing both thiol and propyl silane chains (CNC-mix) were synthesized through aqueous silylation reactions (Figure S2). Thiol-silylated CNCs were obtained using a chosen cellulose-MPTMS mass ratio of 1:0.5 (CNC-thiol I) and 1:1 (CNC-thiol II). The CNC-mix samples were fabricated with both thiol and propyl silane reagents simultaneously as described in a previous study^27^ using the cellulose-MPTMS-PTS mass ratio of 1:0.5:0.5 (CNC-mix I) and 1:1:1 (CNC-mix II). Before silylation reactions, the pH value of CNC suspensions (1% w/w) was adjusted to 4 using a 1 M HCl solution. A fresh silane solution (10% v/v) was prepared by dissolving the desired silane reagent in ethanol while the mixture was mixed with a magnetic stirrer for 10 min. Finally, the silane solution was added dropwise to the aqueous CNC suspension using a micropipet while constantly stirring at room temperature for 2 h. The CNC suspension was then heated at 80 °C for a predefined time of 3 h to favor the condensation reaction and formation of Si–O–cellulose bonds. After the reactions, all of the functionalized CNC samples were washed using dialysis bags and placed in a beaker of 2 L volume to remove nonreacted silanes. The dialysis bags were stirred in a mixture of deionized water and EtOH (7:3 H_2_O/EtOH) for 24 h, followed by stirring in deionized water for four days only. Fresh washing liquid was changed every 24 h.

### X-ray Photoelectron Spectroscopy Analysis and Grafting Amount

An ESCALAB 250Xi X-ray photoelectron spectrometer (Thermo Fisher Scientific, UK) with a monochromatic Al Kα (1486.6 eV) source was used to analyze the carbon, oxygen, silicon, and sulfur contents of the pristine and silylated CNC films (Table S2). The films were prepared by diluting pristine and silylated CNC samples in 40 mL of deionized water to a final consistency of 0.5% w/w and drying them overnight at 50 °C. The grafting amount (GA) of thiol silane groups (mmol_reagent_/g_CNC_) was calculated based on the sulfur content of CNCs-thiol and CNCs-mix, whereas GA of propyl silane groups was calculated from silicon content.^27^

### ζ Potential Analysis

The ζ potential was determined for aqueous suspensions of pristine and silylated cellulose nanocrystals, pure minerals, and mixed suspensions containing pure minerals conditioned with CNC-thiol II at pH values of 4, 6, 9, and 11 using a Malvern ZetaSizer Pro (Malvern Panalytical, UK) based on electrophoretic mobility measurement. A 10 mM NaCl background solution with a preadjusted pH value was used for all samples. The CNC suspensions were diluted by adding 100 μL of 0.1% w/w aqueous CNC sample to 10 mL of a background solution, while the pure mineral dispersions were prepared by adding 10 mg of mineral to 10 mL of a background solution. Finally, the mixed solutions were obtained by adding 10 mg of pure minerals to 10 mL of a background solution, followed by 100 μL of a 0.1% w/w aqueous CNC-thiol II sample. After the minerals were added, the pH was readjusted to desired levels using NaOH or HCl. The data were collected at room temperature, and six measurements were performed for each sample.

### Contact Angle Measurement

The static contact angles of sessile droplets on pristine and silylated cellulose nanocrystal films were measured using a drop shape analyzer (DSA 25 Krüss, Germany). To prepare cellulose films, the CNC samples were diluted in 40 mL of deionized water to a final consistency of 0.5% (w/w) and then dried in an oven at 50 °C to obtain self-standing films. A drop (8 μL) of deionized water was placed on the cellulose film surface to measure the static contact angle. Six droplets were placed in different locations on the cellulose film for each sample and analyzed by using the ellipse-fitting model, which can measure the contact angle of a nonasymmetric drop.

Captive bubble measurements were conducted using flat mineral specimens (chalcopyrite and pyrite) of appropriate size and surface topology, immersed into an aqueous phase, and placed on two stands with the functional surface arranged horizontally facing the bottom of a transparent container. The mineral surface must be partially wetted by water to prevent a thin aqueous film from hindering the attachment of the gas bubble to the specimen surface and obstructing the formation of a three-phase contact line. The specimens were conditioned in 25 mL of 10 mM NaCl solution with a preadjusted pH value of 4, 6, 9, and 11. In each case, 100 μL of either SIBX 0.1% w/w or CNC-thiol II 0.1% w/w was added separately to the NaCl solution and stirred for 10 min. The simultaneous effect of the depressant and collector was analyzed by adding first CNC-thiol II (100 μL) for 10 min under stirring, followed by SIBX (100 μL) and conditioning for 10 min. Finally, the specimens were rinsed with deionized water and placed on a holder in the background solution. A small air bubble with a volume of 8 μL was attached to the bottom of the flat specimen immersed in 25 mL of the background liquid, and the contact angle was recorded at six different locations of the flat surface using a drop shape analyzer (DSA 25 Krüss, Germany). For each new measurement, the specimens were properly polished to avoid any oxidation or contamination and ensure an appropriate smoothness of the surface using a small drop of diamond paste (1 μm) on a velvet cloth and subsequently rinsed twice with EtOH and H_2_O.

### Transmission Electron Microscopy

Transmission electron microscopy (TEM) analysis was used to visualize the structure of the silylated CNCs using a Tecnai G2 Spirit 120 kV instrument (Thermo Fisher Scientific, UK). The silylated CNC suspensions were placed on a sample holder by using a carbon-coated copper grid. A small droplet of 0.1% w/w poly-l-lysine suspension was added to the top of the grid, and the excess was removed by touching the corner of a filter paper. The CNC suspensions were first diluted with deionized water, and then, a small droplet was poured onto the grid’s surface. Finally, a 2% (w/w) uranyl acetate solution was dropped to negatively stain the sample; the excess was removed with the corner of a filter paper. The samples were dried overnight at room temperature and analyzed under standard conditions using an acceleration voltage of 100 kV. Images were captured using a Quemesa camera (Olympus Soft Imaging Solutions GMBH, Münster, Germany), and the length and width of individual cellulose nanocrystals were measured using ImageJ software.

### Field Emission Scanning Electron Microscopy

FESEM Ultra (Zeiss Ultra Plus Oberkochen, Germany) with energy dispersive spectroscopy (EDS) analysis was used to image and address the variation in the elemental composition of pure chalcopyrite and pyrite surface after reacting with CNC-thiol II. Initially, the surface of minerals (chalcopyrite or pyrite) was gently refreshed in a porcelain mortar by grinding for 1 min; then, 1 g of the pure mineral was added in 100 mL of 10 mM NaCl background solution at pH 6 and stirred for 1 min. CNC-thiol II solution (0.1% w/w) was then added to obtain a final concentration of 2.5 mg/L, and the slurry was stirred for another 5 min. After the second conditioning, the minerals were vacuum filtered, rinsed with 100 mL of background solution, and dried at 50 °C overnight. The mineral powder was deposited onto a carbon tape and coated with carbon before imaging with a 15 kV electron high tension.

### Microflotation Tests

Microflotation experiments with single minerals were performed in an in-house-built Hallimond tube (200 mL). The mineral floatability was tested with pure minerals (1 g), then with SIBX as a collector, and simultaneously with an SIBX collector and pristine and silylated CNCs as depressants as a function of pH (4, 6, 9, and 11), and finally with different concentrations of CNC-thiol II (2.5, 5, 10, 15, and 20 mg/L). Initially, the surface of the mineral was gently polished in a porcelain mortar through grinding for 1 min to remove any superficial oxidation. The mineral powder was then dispersed into 100 mL of a 10 mM NaCl background solution with a predefined pH value and stirred for 1 min. To investigate the effect of the collector, a 0.1% w/w SIBX solution was added to the dispersion after mineral conditioning to obtain a concentration of 2.5 mg/L, and the mixture was stirred for 5 min. The simultaneous effect of collector and depressant was analyzed by adding 0.1% w/w CNCs solution to the dispersion after mineral conditioning to obtain concentrations of 2.5, 5, 10, 15, and 20 mg/L and stirring for 5 min, followed by the addition of 0.1% w/w SIBX solution (2.5 mg/L) and stirring for another 5 min. After completing each conditioning, the slurry was transferred into the Hallimond tube and adjusted to the desired water level with the background solution. For all the flotation tests, compressed air at a flow rate of 50 mL/min and a flotation time of 5 min was used. The collected chalcopyrite and pyrite from the overflow and underflow were separately filtered, dried, and weighed to determine recoveries. Each flotation test was performed in triplicate.

## Results and Discussion

### Aqueous Silylation of CNCs with Thiol and Propyl Silanes

A commercial CNC was grafted in aqueous silylation reactions with MPTMS or simultaneously with MPTMS and PTS to obtain thiol-silylated cellulose nanocrystals or bifunctionalized nanocrystals containing both thiol and propyl silane moieties (Figure S2). The silylation reactions were performed using two different cellulose-to-silane mass ratios at an acidic pH of 4. Previous publications indicate that acidic hydrolysis of MPTMS promotes the formation of reactive silanol groups and reduces the self-condensation reaction of ensuing silanols.^39^ In this study, MPTMS was highly reactive under acidic conditions, with 95% of silanes hydrolyzed to silanol groups after a 2 h reaction. In addition, silane solution concentrations of 10% w/w facilitated silanol formation and reduced self-condensation.^35^ The dissolution of silane first in ethanol further increased its solubility in the aqueous reaction medium and ensured uniform coverage of the reagent on the CNC substrate.^40^

### Characterization of Silylated CNCs: TEM, XPS, Contact Angle, and Electrophoretic Mobility

According to TEM analysis, the pristine CNC consisted of typical rod-like cellulose nanocrystals with an average width of 3–8 nm and a length of 50–350 nm.^41^ The functionalized CNCs maintained their morphology and were similar in size, with an average width of 3–6 nm and a length of 250–360 nm (Figure S3). The grafting amount (GA) of silanes on CNCs was determined by using X-ray photoelectron spectroscopy by analyzing the elemental contents of silicon and sulfur in pristine and silylated CNCs (Table S2). The increase in the mass ratio of silylation agents, such as MPTMS and PTS promoted the grafting reaction, and both CNC-thiol II and CNC-mix II had a total GA of >1.5 mmol/g in terms of Si content. This increase in GA was also noted in the sulfur content of thiol-silylated CNCs, and the sulfur amount increased from 0.22 to 1.05 mmol/g (CNC-thiol I vs CNC-thiol II), respectively, when the mass ratio of MPTMS increased from 1:0.5 to1:1. However, the low sulfur content of bifunctionalized CNC at higher silylation reagent mass ratios (CNC-mix II) indicated that the simultaneous silylation reaction with MPTMS and PTS was dominated by the PTS; i.e., the CNC-mix was mainly grafted by alkyl silanes. This difference could be attributed to a higher degree of MPTMS self-condensation. In addition, the total silane content of CNC-mix II was only slightly higher than that of CNC-thiol II (1.67 vs 1.59 mmol/g), indicating that the initial mass ratio of silanes was probably too high for efficient bifunctionalization, resulting in a more pronounced self- or heterocondensation of silanols.

The static contact angles showed that the hydrophobicity of the functionalized CNCs increased as silane and sulfur concentrations increased (Table S3). CNC-thiol II, with a higher content of silanes, also exhibited a higher static contact angle value of 68.6° (Table S3), whereas CNC-thiol I (approximately 38.0°) had a lower value. CNC-mix II had a contact angle of 52.5°, indicating that the higher amount of propyl silane promoted the formation of a more homogeneous cellulose film and decreased the surface roughness. Overall, the contact angle for all functionalized CNCs remained relatively low (<70°).

Throughout the examined conditions (pH range 4–11), all CNCs exhibited a negative electric surface ζ-potential, which was only slightly influenced by the silylation reactions (Figure S4). This could be due to the relatively low proportion of pH-responsive SH groups on CNC surfaces compared with surface hydroxyl groups, and their moderately low acidity (despite the acidity being higher than that of hydroxyls in pristine cellulose). Overall, the ζ-potential of samples behaved similarly as a function of pH, with the ζ-potential becoming more negative at alkaline conditions due to deprotonation and less than −25 mV at pH 11 for all CNCs.

### Flotation of Chalcopyrite and Pyrite Using Silylated CNCs as Depressants

**Figure 1:**
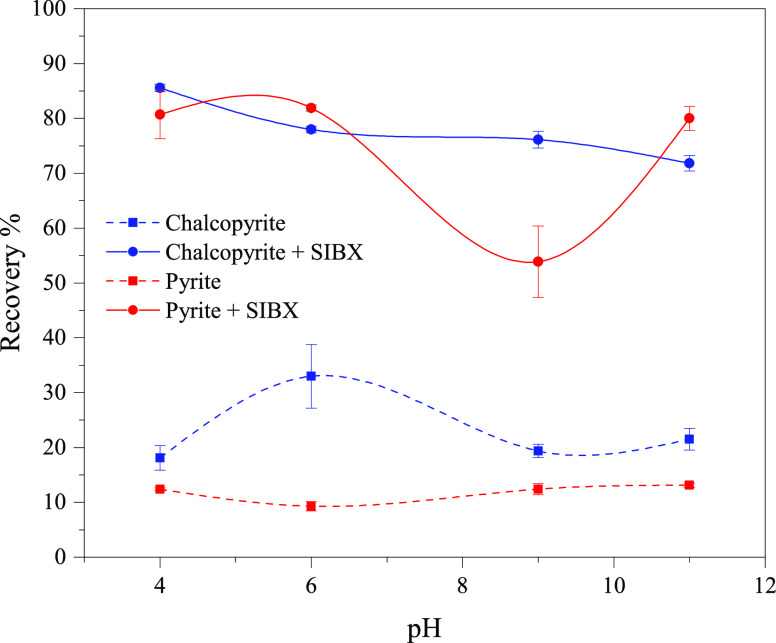
Flotation recovery of chalcopyrite and pyrite in the absence and presence of SIBX as a collector (2.5 mg/L); error bars represent standard deviations (*n* = 3).

The performance of silylated CNCs as depressants in the flotation of chalcopyrite and pyrite was elucidated using an in-house built microflotation cell. First, the floatability of single mineral suspensions was investigated in terms of flotation recovery as a function of pH values (4, 6, 9, and 11) in the presence and absence of a commercial SIBX collector (Figure 1). Both minerals exhibited inherently poor floatability without the collector throughout the entire pH range, with an average recovery of ∼20% for chalcopyrite and ∼10% for pyrite. The maximum recovery of 33% was obtained with chalcopyrite at pH 6. The SIBX collector improved the recovery of both chalcopyrite and pyrite significantly, and the maximum recovery (86% for chalcopyrite and 93% for pyrite) was reached at pH 4. SIBX can promote the floatability of sulfidic minerals efficiently, despite its limited selectivity, and as demonstrated in this study, it cannot separate chalcopyrite and pyrite selectively from their mixtures because of their similar recovery.^30−32^ One approach is to use high amounts of lime to depress pyrite at pH values well above 10 or by the depression of chalcopyrite, as suggested in this study.

The mineral floatability was further elucidated using pristine and silylated CNCs as depressants in the presence of SIBX as a collector. The CNC concentration during the conditioning of the minerals was 10 mg/L, while the concentration of SIBX was kept constant at 2.5 mg/L throughout all experiments. Microflotation tests were performed at pH 4, resulting in high recovery with both minerals in the presence of SIBX (without CNCs Figure 1), and at pH 9, promoting the recovery of chalcopyrite, which is the most promising condition for the selective flotation of chalcopyrite (Figure 1).

**Figure 2:**
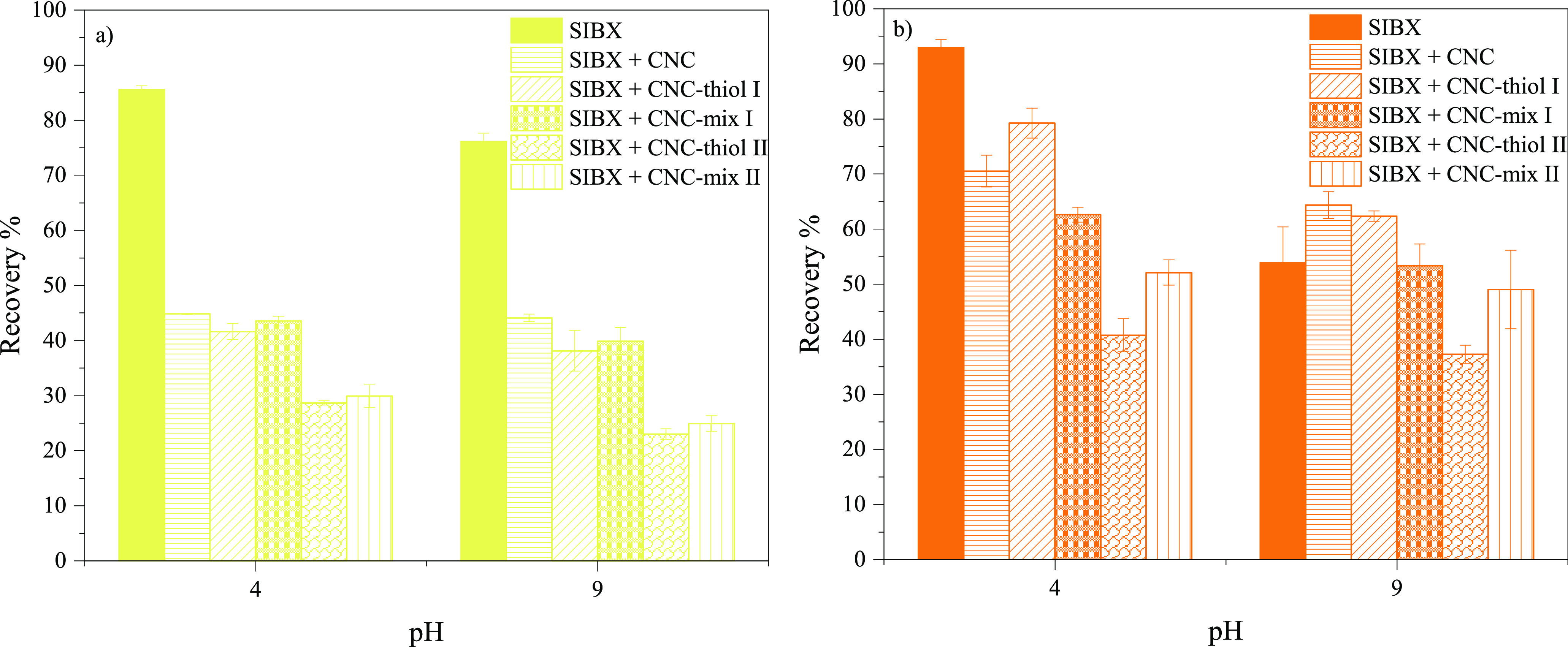
Flotation recovery of (a) chalcopyrite and (b) pyrite in the presence of CNCs and SIBX; error bars represent standard deviations (*n* = 3).

Figure 2 shows that the addition of both pristine and silylated CNCs significantly decreased the overall recovery of both minerals, with chalcopyrite showing the highest decrease in floatability. However, in the presence of pristine CNC and CNC-thiol I, pyrite recovery increased unexpectedly at pH 9 (the recoveries being 64% and 62% vs 54%, respectively). This anomaly could be explained by poor CNC interaction and adsorption on the pyrite surface at high pH, as well as more pronounced bubble formation due to unabsorbed CNC. Generally, silylated CNCs with a higher degree of substitution, such as CNC-thiol II and CNC-mix II, demonstrated a higher depressing efficiency toward both chalcopyrite and pyrite, whereas the pristine CNC and CNCs with a lower GA, such as CNC-thiol I and CNC-mix I, demonstrated a lower and similar performance.

**Figure 3:**
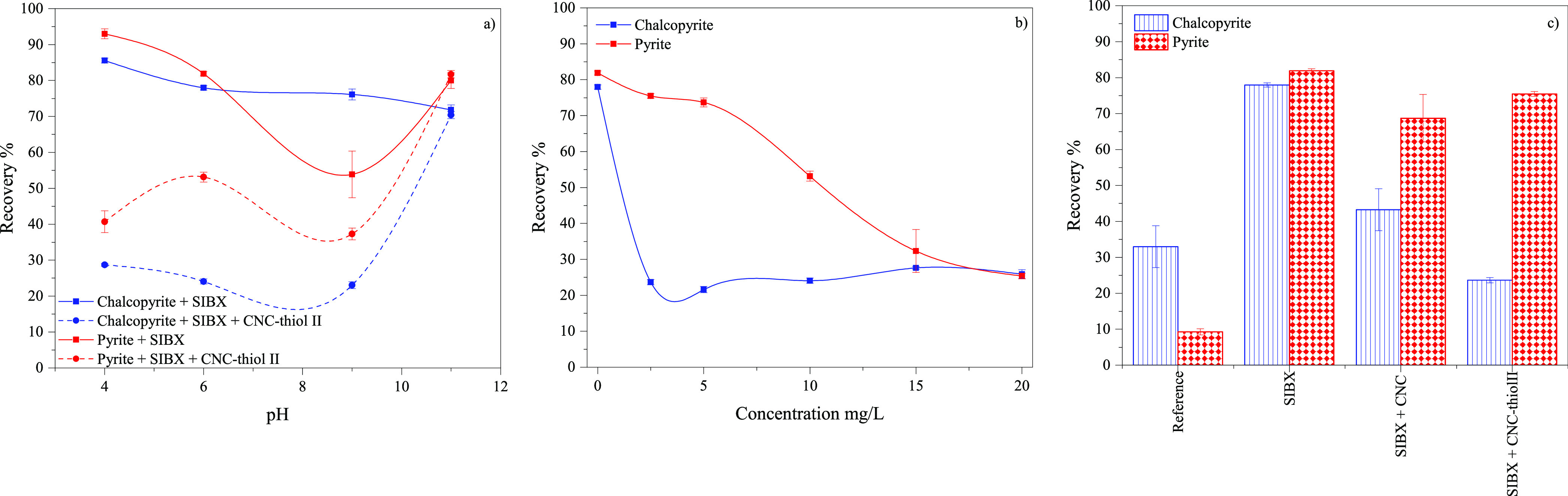
Flotation recovery of chalcopyrite and pyrite (a) as a function of pH in the presence or absence of CNC-thiol II as depressant and SIBX as a collector, (b) as a function of CNC-thiol II concentration at pH 6, and (c) in the absence (reference) and presence of SIBX, CNC, and CNC-thiol II at pH of 6 and at a concentration of 2.5 mg/L; error bars represent standard deviations (*n* = 3).

The performance of CNC-thiol II, which demonstrated the most efficient depression activity among the synthesized CNCs, was further revealed as a function of pH (4, 6, 9, and 11) at a constant CNC concentration of 10 mg/L (Figure 3a). CNC-thiol II substantially affected the floatability of both minerals below pH 9, with chalcopyrite and pyrite recovering at ∼25% and 44%, respectively, indicating a general depression of these minerals. At a high pH of 11, the role of CNC-thiol II in mineral recovery was negligible, implying that the interaction between the mineral surfaces and silylated CNCs was poor, which could be due to the charge repulsion caused by the high negative surface charge density of both constituents. Generally, thiol-silylated CNC (CNC-thiol II) demonstrated a stronger depression efficiency with chalcopyrite under specific conditions, implying its potential to promote the selectivity of chalcopyrite/pyrite flotation.

The dependency of the flotationability of chalcopyrite and pyrite on the concentration of CNC-thiol II was investigated at a constant pH of 6 (Figure 3b). The recovery of the minerals differed substantially as a function of CNC dosage, and a drastic decrease in chalcopyrite floatability was observed at a low CNC dosage of 2.5 mg/L. Its recovery remained low at approximately 25%–30%. In turn, the recovery of pyrite decreased continuously with CNC-thiol II concentration, reaching a low recovery of 25% at a CNC dosage of 20 mg/L. Consequently, the depressant efficiency of CNC-thiol II toward chalcopyrite was significantly higher than that of pyrite, demonstrating the potential of silylated CNCs for selective depression of chalcopyrite under specific conditions.

To confirm the actual efficiency of CNC-thiol II as a depressant toward chalcopyrite and pyrite under appropriate flotation process conditions, mineral recoveries were determined at an optimal CNC concentration of 2.5 mg/L at pH 6 (Figure 3c) and compared with the performance of pristine CNC. The flotation selectivity in terms of the difference in chalcopyrite and pyrite recovery was 3.9% without any CNC, 25.5% with pristine CNC, and 51.8% with CNC-thiol II. Therefore, the nonmodified CNC exhibited an inherent attraction on the surface of the mineral and pronounced depression activity toward chalcopyrite. The presence of a thiol moiety grafted onto the cellulose surface further promoted this selectivity. Overall, thiol-silylated CNC had a better affinity and interaction with chalcopyrite than pyrite, and the silylation significantly increased selectivity. In summary, both the characteristics of CNC and the processing conditions were crucial in obtaining a selective depression of chalcopyrite. A maximum difference in the recovery of chalcopyrite and pyrite of ∼52% was obtained with a CNC-thiol II and SIBX concentration of 2.5 mg/L at a pH value of 6, indicating the potential of CNC-thiol II as a selective depressant for chalcopyrite.

In previous studies, thiol groups containing chemicals, such as pseudo-glycolythiourea acid (PGA), tiopronin, and dithiothreitol (DTT) have been used to selectively depress chalcopyrite.^24,42,43^ Thiol groups can absorb chemically on the chalcopyrite surface, while the hydrophilic moieties of chemicals protrude toward the water interface. Thiol groups were covalently linked with the copper sites on the chalcopyrite surface when using DTT and PGA,^24,43^ whereas tiopronin formed a five-membered chelating ring via carbonyl and thiol groups bonded with the copper atoms into the chalcopyrite.^42^ Therefore, the interactions between thiol groups grafted onto the cellulose and mineral surfaces enhanced the selective depression of chalcopyrite with CNC-thiol II. Meanwhile, the hydroxyl groups of adsorbed CNC are directed toward the aqueous phase, resulting in a highly hydrophilic chalcopyrite surface. However, further studies will be required to investigate the preferential interaction of thiol-silylated CNC-thiol II with the chalcopyrite surface.

### Wettability of Minerals in Presence of Propyl Silane CNC

Static contact angle measurements have traditionally been used to assess and predict the floatability of minerals, although wetting behavior may not realistically indicate the actual performance of the flotation process. The contact angle is a thermodynamic quantity obtained under equilibrium conditions, whereas flotation is a highly dynamic process. In addition, other factors may affect the flotation, such as the physical and mechanical features of thinning liquid films upon collision, the hydrodynamics of mineral–water suspensions, and the orthokinetic attachment of mineral particles to the air bubble.^44^ In this study, a captive bubble method, which reflects the behavior of a complex flotation system better than the static sessile-drop technique, was used to analyze the wettability of minerals under aqueous conditions, although a flat mineral surface used in the captive bubble measurement does not exactly represent individual particles.^45^

**Figure 4:**
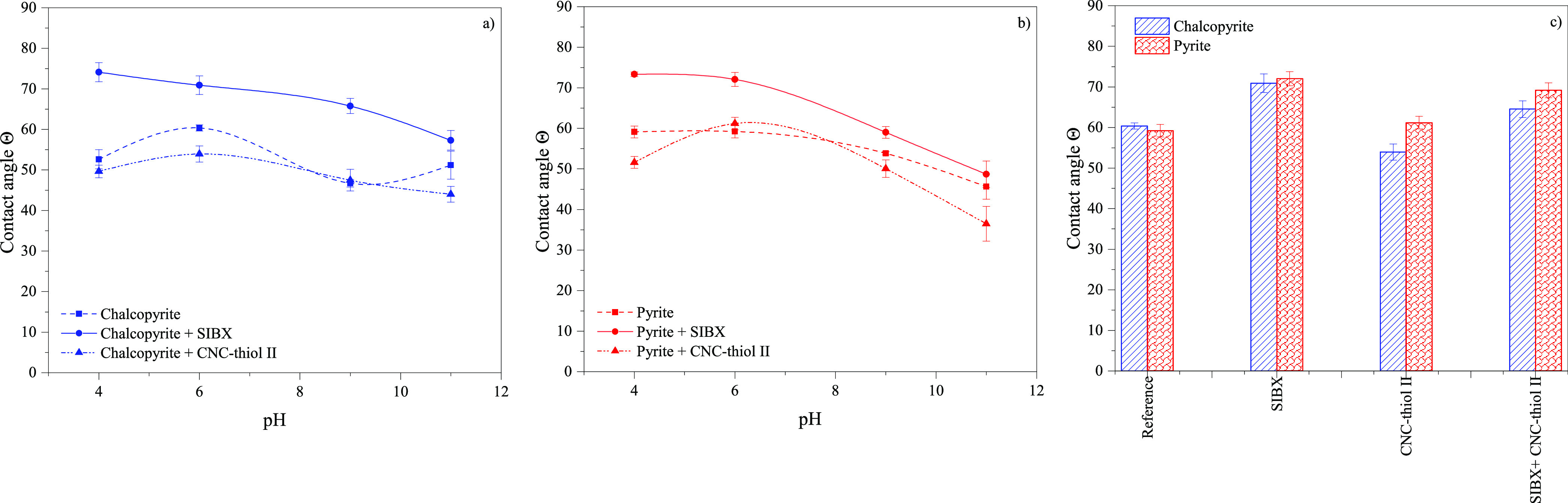
Contact angle (captive bubble method) of pure mineral specimens: (a) chalcopyrite, (b) pyrite after conditioning with SIBX or CNC-thiol II, and (c) after conditioning with SIBX or CNC-thiol II, respectively, at pH of 6; error bars represent standard deviations (*n* = 6).

Minerals are commonly classified as either polar (naturally hydrophilic), which interact strongly with water molecules, or nonpolar (inherently hydrophobic with contact angles above 60°), which do not readily interact with water molecules. Sulfide minerals such as chalcopyrite and pyrite are commonly categorized as naturally hydrophobic minerals.^16^ However, in this study, both chalcopyrite and pyrite exhibited a low hydrophobicity of approximately 60° (Figure 4a, b), and this relatively high polarity was reflected in the low inherent floatability of both minerals in microflotation (Figure 1). The SIBX collector significantly increased the hydrophobicity of both minerals, with the maximum contact angle being 74.1° for chalcopyrite and 73.3° for pyrite at pH 4, respectively. The addition of CNC-thiol II significantly decreased the contact angle values of both minerals, inferring that silylated CNC interacted with mineral surfaces and decreased their hydrophobic character.

Figure 4c shows the contact angle of pristine minerals before and after separately or simultaneously adding the SIBX collector and CNC-thiol II into the conditioning solution at an optimum flotation pH of 6 (Figure 3a). Chalcopyrite exhibited a systematically lower contact angle under all conditions except for the reference (pure minerals), and this difference in contact angle values was increased when thiol-silylated CNC was dosed in the solution. This behavior indicated the preferential adsorption and interaction of CNC-thiol II with chalcopyrite, supporting the findings on reduced floatability of minerals in the presence of thiol-silylated CNC. However, the difference in contact angles of minerals was significantly smaller than the difference in floatability (compare with Figure 3c), indicating that other factors could be responsible for the recovery of chalcopyrite and pyrite or that the contact angle measurement did not accurately reflect the conditions during microflotation. This highlights the complex morphology and heterogeneous surface chemistry of CNCs and minerals. For example, the contact angle measurements were performed under quasistatic conditions, in which the grafted silanes may orient themselves toward more favorable phases, i.e., so that the hydrophobic propyl groups protrude toward the air bubble and the relatively hydrophilic thiol groups toward the mineral surface, thus the grafting may slightly affect only the surface wettability.^46,47^ Moreover, the CNC may form a heterogeneous and patchy-like coating on the mineral surface, which is difficult to measure using the contact angle method.

### Influence of Thiol Silane CNCs on ζ-Potential of Mineral Suspensions

**Figure 5:**
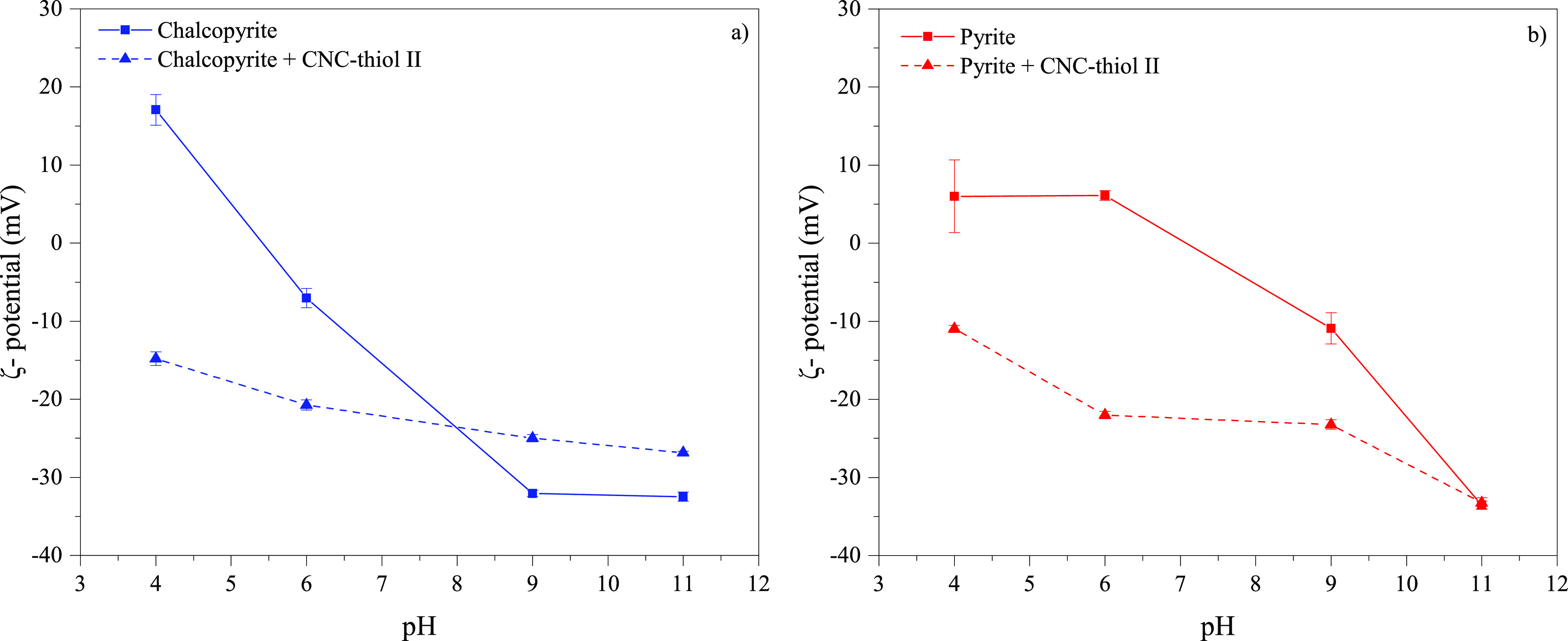
ζ-potential of (a) pure chalcopyrite and (b) pyrite and after the addition of CNC-thiol II, as a function of the pH value; error bars represent standard deviations (*n* = 6).

Figure 5 shows the ζ-potentials of chalcopyrite and pyrite particles before and after reacting with CNC-thiol II. Chalcopyrite shows a positive ζ-potential (∼18 mV) at an acidic pH of 4, and it decreases sharply and almost linearly to a constant level of approximately −32 mV at pH 9. The addition of CNC-thiol II significantly reduced the ζ-potential at a pH range from 4 (+17 vs −14.8 mV) to 6 (−7.0 vs −20.7 mV), while the change in surface charge caused by CNC is only minor under alkaline conditions. CNC-thiol II shifts the initial ζ-potential toward less negative values at pH ranging from 9 (−32.1 vs −24.1 mV) to 11 (−32.5 vs −26.9 mV). Under alkaline conditions, the small ζ-potential difference between pure and CNC-thiol II-coated chalcopyrite indicates that CNC adsorption on the chalcopyrite surface is low. This finding correlates with the flotation results shown in Figure 3a. However, pure pyrite exhibited some differences in its surface charge behavior, with the ζ-potential being more stable at acidic pH and the isoelectric point being at pH of ∼7. Notably, both minerals have similar ζ-potentials in the presence of CNC-thiol II, except at a highly alkaline pH of 11.

### Morphology and Elemental Composition of Mineral Surfaces in the Presence of Thiol Silane CNCs

The surface structure and elemental composition of individual chalcopyrite and pyrite particles were revealed via FESEM and EDS analyses to directly detect the presence of CNC on the mineral surfaces. Figure S5 shows the visual appearance of mineral particles before and after conditioning with CNC-thiol II and the five different locations used for elemental mapping (Table S4). After conditioning of mineral with CNC-thiol II, a significant decrease in Cu and S contents (attributed to the elements of chalcopyrite) and an increase in Si content (attributed to the silylated CNC) were observed, indicating the presence of silylated cellulose nanocrystals on the mineral surface. The large variation in the elemental contents and standard deviations suggested that CNC-thiol II was unevenly distributed on the mineral surface, forming hydrophilic patches on the chalcopyrite surface, as supported by the flotation and contact angle results (Figures 3c and 4a). This inhomogeneous adsorption was probably due to variations in adsorption sites on the mineral surface, differences in cleavage surfaces produced during particle crushing and grinding,^48^ and the rod-like shape of CNC. The minor changes in the contents of the main elements of pyrite (Fe and S) and the absence of Si (silylated CNC) suggested that the interaction between CNC-thiol II and pyrite was poor or negligible, consistent with the flotation results (Figure 3c) and supporting the preferential adsorption of silylated CNC on chalcopyrite.

## Conclusions

Silylated cellulose nanocrystals, that is, CNCs containing thiol silane groups and bifunctionalized CNCs containing both thiol and propyl silane chains, were synthesized using aqueous silylation reactions, which were demonstrated to be a smart approach to modify the CNCs, combining the use of a renewable resource and sustainability modification reactions. Their performance as depressants in the flotation of chalcopyrite and pyrite in the presence of an SIBX collector was investigated in a microflotation system. Thiol-silylated CNC with the highest degree of grafting (CNC-thiol II) demonstrated the best selectivity toward chalcopyrite depression at a relatively low concentration of 2.5 mg/L, with a 52% difference in chalcopyrite and pyrite recovery, indicating preferential adsorption of silylated CNC on chalcopyrite over pyrite, as confirmed by EDS analysis. In summary, the silylated CNCs provide a new class of sustainable biodepressants that could be used for promoting the flotation selectivity of sulfide minerals. In addition, renewable depressants based on bifunctionalized nanocelluloses can pave the way toward more sustainable mineral processing decreasing the drawbacks related to inorganic and polymeric depressants and enhancing the efficiency and selectivity of ore beneficiation processes. Further studies will be conducted to investigate the efficiency and selectivity of silylated CNCs in mixed mineral systems.

## Supporting Information Available

Table S1, particle diameter of quantile sizes and mean diameter of chalcopyrite and pyrite; Figure S1, particle size distribution of fine and coarse fractions of chalcopyrite and pyrite; Figure S2, synthesis of CNCs containing thiol silane group and CNCs containing thiol and propyl silane groups via aqueous silylation; Figure S3, TEM images of functionalized CNCs; Table S2, composition of pristine and functionalized CNCs from XPS analysis*;* Table S3, grafting amount and static contact angle of pristine and silylated CNCs; Figure S4, ζ-potential of pristine and silylated CNCs; Figure S5, FESEM images of chalcopyrite and pyrite in the presence and absence of CNC-thiol II; Table S4, elemental composition (% w/w) of chalcopyrite and pyrite in presence and absence of CNC-thiol II (PDF)

## References

[ref1] Tang J.; Sisler J.; Grishkewich N.; Tam K. C. Functionalization of Cellulose Nanocrystals for Advanced Applications. J. Colloid Interface Sci. 2017, 494, 397–409. 10.1016/j.jcis.2017.01.077. 28187295

[ref2] Sharma A.; Thakur M.; Bhattacharya M.; Mandal T.; Goswami S. Commercial Application of Cellulose Nano-Composites – A Review. Biotechnol. Rep. 2019, 21, e00316 10.1016/j.btre.2019.e00316. PMC6389799 30847286

[ref3] Aalbers G. J. W.; Boott C. E.; D’Acierno F.; Lewis L.; Ho J.; Michal C. A.; Hamad W. Y.; MacLachlan M. J. Post-Modification of Cellulose Nanocrystal Aerogels with Thiol–Ene Click Chemistry. Biomacromolecules 2019, 20 (7), 2779–2785. 10.1021/acs.biomac.9b00533. 31244013

[ref4] Fatima A.; Yasir S.; Khan M. S.; Manan S.; Ullah M. W.; Ul-Islam M. Plant Extract-Loaded Bacterial Cellulose Composite Membrane for Potential Biomedical Applications. J. Bioresour. Bioprod. 2021, 6 (1), 26–32. 10.1016/j.jobab.2020.11.002.

[ref5] Joseph B.; K S. V.; Sabu C.; Kalarikkal N.; Thomas S. Cellulose Nanocomposites: Fabrication and Biomedical Applications. J. Bioresour. Bioprod. 2020, 5 (4), 223–237. 10.1016/j.jobab.2020.10.001.

[ref6] Jedvert K.; Heinze T. Cellulose Modification and Shaping – a Review. J. Polym. Eng. 2017, 37 (9), 845–860. 10.1515/polyeng-2016-0272.

[ref7] Kalia S.; Dufresne A.; Cherian B. M.; Kaith B. S.; Avérous L.; Njuguna J.; Nassiopoulos E. Cellulose-Based Bio- and Nanocomposites: A Review. Int. J. Polym. Sci. 2011, 2011, 1–35. 10.1155/2011/837875.

[ref8] Kalia S.; Boufi S.; Celli A.; Kango S. Nanofibrillated Cellulose: Surface Modification and Potential Applications. Colloid Polym. Sci. 2014, 292 (1), 5–31. 10.1007/s00396-013-3112-9.

[ref9] Rong L.; Zhu Z.; Wang B.; Mao Z.; Xu H.; Zhang L.; Zhong Y.; Sui X. Facile Fabrication of Thiol-Modified Cellulose Sponges for Adsorption of Hg2+ from Aqueous Solutions. Cellulose 2018, 25 (5), 3025–3035. 10.1007/s10570-018-1758-7.

[ref10] Wu Y.; Zhang Y.; Chen N.; Dai S.; Jiang H.; Wang S. Effects of Amine Loading on the Properties of Cellulose Nanofibrils Aerogel and Its CO2 Capturing Performance. Carbohydr. Polym. 2018, 194, 252–259. 10.1016/j.carbpol.2018.04.017. 29801837

[ref11] Wang Z.; Zhang X.-F.; Ding M.; Yao J. Aminosilane-Modified Wood Sponge for Efficient CO2 Capture. Wood Sci. Technol. 2022, 56 (3), 691–702. 10.1007/s00226-022-01371-4.

[ref12] Coelho Braga de Carvalho A. L.; Ludovici F.; Goldmann D.; Silva A. C.; Liimatainen H. Silylated Thiol-Containing Cellulose Nanofibers as a Bio-Based Flocculation Agent for Ultrafine Mineral Particles of Chalcopyrite and Pyrite. J. Sustain. Metall. 2021, 7 (4), 1506–1522. 10.1007/s40831-021-00439-y.

[ref13] Lopéz R.; Jordão H.; Hartmann R.; Ämmälä A.; Carvalho M. T. Study of Butyl-Amine Nanocrystal Cellulose in the Flotation of Complex Sulphide Ores. Colloids Surf. Physicochem. Eng. Asp. 2019, 579, 123655 10.1016/j.colsurfa.2019.123655.

[ref14] Hartmann R.; Sirviö J. A.; Sliz R.; Laitinen O.; Liimatainen H.; Ämmälä A.; Fabritius T.; Illikainen M. Interactions between Aminated Cellulose Nanocrystals and Quartz: Adsorption and Wettability Studies. Colloids Surf. Physicochem. Eng. Asp. 2016, 489, 207–215. 10.1016/j.colsurfa.2015.10.022.

[ref15] Urbina R. H. Recent Developments and Advances in Formulations and Applications of Chemical Reagents Used in Froth Flotation. Miner. Process. Extr. Metall. Rev. 2003, 24 (2), 139–182. 10.1080/08827500306898.

[ref16] Wills B. A. Chapter 12 - Froth Flotation. In Mineral Processing Technology Fourth ed.; Wills B. A., Ed.; International Series on Materials Science and Technology; Pergamon: Amsterdam, 1988; pp 457–595. 10.1016/B978-0-08-034937-4.50021-1.

[ref17] Laskowski J. S.; Liu Q.; O’Connor C. T. Current Understanding of the Mechanism of Polysaccharide Adsorption at the Mineral/Aqueous Solution Interface. Int. J. Miner. Process. 2007, 84 (1), 59–68. 10.1016/j.minpro.2007.03.006.

[ref18] Yin Z.; Sun W.; Hu Y.; Zhang C.; Guan Q.; Liu R.; Chen P.; Tian M. Utilization of Acetic Acid-[(Hydrazinylthioxomethyl)Thio]-Sodium as a Novel Selective Depressant for Chalcopyrite in the Flotation Separation of Molybdenite. Sep. Purif. Technol. 2017, 179, 248–256. 10.1016/j.seppur.2017.01.049.

[ref19] Pearse M. J. An Overview of the Use of Chemical Reagents in Mineral Processing. Miner. Eng. 2005, 18 (2), 139–149. 10.1016/j.mineng.2004.09.015.

[ref20] Ansari A.; Pawlik M. Floatability of Chalcopyrite and Molybdenite in the Presence of Lignosulfonates. Part II. Hallimond Tube Flotation. Miner. Eng. 2007, 20 (6), 609–616. 10.1016/j.mineng.2006.12.008.

[ref21] Yin Z.; Sun W.; Hu Y.; Zhai J.; Qingjun G. Evaluation of the Replacement of NaCN with Depressant Mixtures in the Separation of Copper–Molybdenum Sulphide Ore by Flotation. Sep. Purif. Technol. 2017, 173, 9–16. 10.1016/j.seppur.2016.09.011.

[ref22] Mu Y.; Peng Y.; Lauten R. A. The Depression of Pyrite in Selective Flotation by Different Reagent Systems – A Literature Review. Miner. Eng. 2016, 96–97, 143–156. 10.1016/j.mineng.2016.06.018.

[ref23] Liu Y.; Liu Q. Flotation Separation of Carbonate from Sulfide Minerals, II: Mechanisms of Flotation Depression of Sulfide Minerals by Thioglycollic Acid and Citric Acid. Miner. Eng. 2004, 17 (7), 865–878. 10.1016/j.mineng.2004.03.007.

[ref24] Chen J.; Lan L.; Liao X. Depression Effect of Pseudo Glycolythiourea Acid in Flotation Separation of Copper–Molybdenum. Trans. Nonferrous Met. Soc. China 2013, 23 (3), 824–831. 10.1016/S1003-6326(13)62535-2.

[ref25] Li M.; Wei D.; Shen Y.; Liu W.; Gao S.; Liang G. Selective Depression Effect in Flotation Separation of Copper–Molybdenum Sulfides Using 2,3-Disulfanylbutanedioic Acid. Trans. Nonferrous Met. Soc. China 2015, 25 (9), 3126–3132. 10.1016/S1003-6326(15)63942-5.

[ref26] Khoso S. A.; Hu Y.; Tian M.; Gao Z.; Sun W. Evaluation of Green Synthetic Depressants for Sulfide Flotation: Synthesis, Characterization and Floatation Performance to Pyrite and Chalcopyrite. Sep. Purif. Technol. 2021, 259, 118138 10.1016/j.seppur.2020.118138.

[ref27] Ludovici F.; Hartmann R.; Liimatainen H. Aqueous Bifunctionalization of Cellulose Nanocrystals through Amino and Alkyl Silylation: Functionalization, Characterization, and Performance of Nanocrystals in Quartz Microflotation. Cellulose 2023, 30 (2), 775–787. 10.1007/s10570-022-04961-4.

[ref28] Hartmann R.; Rinne T.; Serna-Guerrero R. On the Colloidal Behavior of Cellulose Nanocrystals as a Hydrophobization Reagent for Mineral Particles. Langmuir 2021, 37 (7), 2322–2333. 10.1021/acs.langmuir.0c03131. 33544605 PMC8023700

[ref29] Panda S.; Akcil A.; Pradhan N.; Deveci H. Current Scenario of Chalcopyrite Bioleaching: A Review on the Recent Advances to Its Heap-Leach Technology. Bioresour. Technol. 2015, 196, 694–706. 10.1016/j.biortech.2015.08.064. 26318845

[ref30] Ma X.; Xia L.; Wang S.; Zhong H.; Jia H. Structural Modification of Xanthate Collectors To Enhance the Flotation Selectivity of Chalcopyrite. Ind. Eng. Chem. Res. 2017, 56 (21), 6307–6316. 10.1021/acs.iecr.6b04566.

[ref31] Huang X.; Huang K.; Jia Y.; Wang S.; Cao Z.; Zhong H. Investigating the Selectivity of a Xanthate Derivative for the Flotation Separation of Chalcopyrite from Pyrite. Chem. Eng. Sci. 2019, 205, 220–229. 10.1016/j.ces.2019.04.051.

[ref32] Jia Y.; Huang K.; Wang S.; Cao Z.; Zhong H. The Selective Flotation Behavior and Adsorption Mechanism of Thiohexanamide to Chalcopyrite. Miner. Eng. 2019, 137, 187–199. 10.1016/j.mineng.2019.04.015.

[ref33] Adrianto L. R.; Ciacci L.; Pfister S.; Hellweg S. Toward Sustainable Reprocessing and Valorization of Sulfidic Copper Tailings: Scenarios and Prospective LCA. Sci. Total Environ. 2023, 871, 162038 10.1016/j.scitotenv.2023.162038. 36740057

[ref34] Adrianto L. R.; Pfister S. Prospective Environmental Assessment of Reprocessing and Valorization Alternatives for Sulfidic Copper Tailings. Resour. Conserv. Recycl. 2022, 186, 106567 10.1016/j.resconrec.2022.106567.

[ref35] Brochier Salon M.-C.; Bayle P.-A.; Abdelmouleh M.; Boufi S.; Belgacem M. N. Kinetics of Hydrolysis and Self Condensation Reactions of Silanes by NMR Spectroscopy. Colloids Surf. Physicochem. Eng. Asp. 2008, 312 (2), 83–91. 10.1016/j.colsurfa.2007.06.028.

[ref36] Abdelmouleh M.; Boufi S.; ben Salah A.; Belgacem M. N.; Gandini A. Interaction of Silane Coupling Agents with Cellulose. Langmuir 2002, 18 (8), 3203–3208. 10.1021/la011657g.

[ref37] Salon M.-C. B.; Gerbaud G.; Abdelmouleh M.; Bruzzese C.; Boufi S.; Belgacem M. N. Studies of Interactions between Silane Coupling Agents and Cellulose Fibers with Liquid and Solid-State NMR. Magn. Reson. Chem. 2007, 45 (6), 473–483. 10.1002/mrc.1994. 17431857

[ref38] Onwukamike K. N.; Grelier S.; Grau E.; Cramail H.; Meier M. A. R. Critical Review on Sustainable Homogeneous Cellulose Modification: Why Renewability Is Not Enough. ACS Sustain. Chem. Eng. 2019, 7 (2), 1826–1840. 10.1021/acssuschemeng.8b04990.

[ref39] Bel-Hassen R.; Boufi S.; Salon M.-C. B.; Abdelmouleh M.; Belgacem M. N. Adsorption of Silane onto Cellulose Fibers. II. The Effect of pH on Silane Hydrolysis, Condensation, and Adsorption Behavior. J. Appl. Polym. Sci. 2008, 108 (3), 1958–1968. 10.1002/app.27488.

[ref40] Brochier Salon M.-C.; Abdelmouleh M.; Boufi S.; Belgacem M. N.; Gandini A. Silane Adsorption onto Cellulose Fibers: Hydrolysis and Condensation Reactions. J. Colloid Interface Sci. 2005, 289 (1), 249–261. 10.1016/j.jcis.2005.03.070. 15907861

[ref41] Laitinen O.; Ojala J.; Sirviö J. A.; Liimatainen H. Sustainable Stabilization of Oil in Water Emulsions by Cellulose Nanocrystals Synthesized from Deep Eutectic Solvents. Cellulose 2017, 24 (4), 1679–1689. 10.1007/s10570-017-1226-9.

[ref42] Yang B.; Yan H.; Zeng M.; Zhu H. Tiopronin as a Novel Copper Depressant for the Selective Flotation Separation of Chalcopyrite and Molybdenite. Sep. Purif. Technol. 2021, 266, 118576 10.1016/j.seppur.2021.118576.

[ref43] Yan H.; Yang B.; Zhu H.; Huang P.; Hu Y. Selective Flotation of Cu-Mo Sulfides Using Dithiothreitol as an Environmental-Friendly Depressant. Miner. Eng. 2021, 168, 106929 10.1016/j.mineng.2021.106929.

[ref44] Somasundaran P. Reagents in Mineral Technology; Routledge, 2018.

[ref45] Drelich J. W.; Marmur A. Meaningful Contact Angles in Flotation Systems: Critical Analysis and Recommendations. Surf. Innov. 2017, 1–45. 10.1680/jsuin.17.00037.

[ref46] Hartmann R.; Rudolph M.; Ämmälä A.; Illikainen M. The Action of Cellulose-Based and Conventional Flotation Reagents under Dry and Wet Conditions Correlating Inverse Gas Chromatography to Microflotation Studies. Miner. Eng. 2017, 114, 17–25. 10.1016/j.mineng.2017.09.004.

[ref47] Hartmann R.; Kinnunen P.; Illikainen M. Cellulose-Mineral Interactions Based on the DLVO Theory and Their Correlation with Flotability. Miner. Eng. 2018, 122, 44–52. 10.1016/j.mineng.2018.03.023.

[ref48] Bai X.; Liu J.; Feng Q.; Wen S.; Dong W.; Lin Y. Study on Selective Adsorption of Organic Depressant on Chalcopyrite and Pyrite Surfaces. Colloids Surf. Physicochem. Eng. Asp. 2021, 627, 127210 10.1016/j.colsurfa.2021.127210.

