# Supplementary material for: Thiol-Silylated Cellulose Nanocrystals as Selective Biodepressants in Froth Flotation
Source: ACS Sustain Chem Eng. 2023 Nov 2;11(45):16176–84. doi: 10.1021/acssuschemeng.3c04013 (PMC10647933; doi:10.1021/acssuschemeng.3c04013)
Supplement: Supplementary file 1 — sc3c04013_si_001.pdf [file sc3c04013_si_001.pdf]

Supporting Information

Thiol-Silylated Cellulose Nanocrystals as Selective Biodepressants in Froth Flotation

Feliciana Ludovici[1†], Robert Hartmann[2‡], Martin Rudolph[3†,‡], Henrikki Liimatainen[1*]

[1†] *Fiber and Particle Engineering Research Unit, University of Oulu, P. O. Box 4300, FI-90014 Oulu, Finland*

[2‡] *Fraunhofer Center for Chemical-Biotechnological Processes, 06237 Leuna, Germany*

[3†,‡] *Helmholtz-Zentrum-Dresden-Rossendorf, Helmholtz Institute Freiberg for Resource Technology, 09599 Freiberg, Germany*

*Corresponding author e-mail: henrikki.liimatainen@oulu.fi

1. Number of Pages: 5 (S1, S2, S3, S4, S5)
2. Number of Figures: 5
3. Number of Tables: 4
4. Page S1: Cover
5. Page S2: Table S1 particle diameter of chalcopyrite and pyrite, Figure S1 particle size distribution of chalcopyrite and pyrite, Figure S2 CNCs modification reaction scheme,
6. Page S3: Figure S3 TEM of modified CNCs, Table S2 XPS analyses of CNCs samples, Table S3 grafting amount and contact angles of CNCs samples,
7. Page S4: Figure S4 ζ-potential of CNCs samples, Figure S5 FESEM images of chalcopyrite and pyrite,
8. Page S5: Table S4 elemental composition (% w/w) of chalcopyrite and pyrite

Table S1. Particle diameter of quantile sizes and mean diameter of chalcopyrite and pyrite.

Size quantiles	Chalcopyrite		Pyrite	
	Fine	Coarse	Fine	Coarse
D_{10}/µm	1.3 ± 0.1	69.7 ± 0.2	2.1 ± 0.1	73.2 ± 0.1
D_{50}/µm	3.4 ± 0.2	116.8 ± 0.3	4.7 ± 0.2	121.9 ± 0.2
D_{90}/µm	12.7 ± 0.7	169.9 ± 0.4	50.3 ± 2.5	187.4 ± 0.5
Mean/µm	5.1 ± 0.6	116.2 ± 0.2	16.2 ± 0.3	125.8 ± 0.1

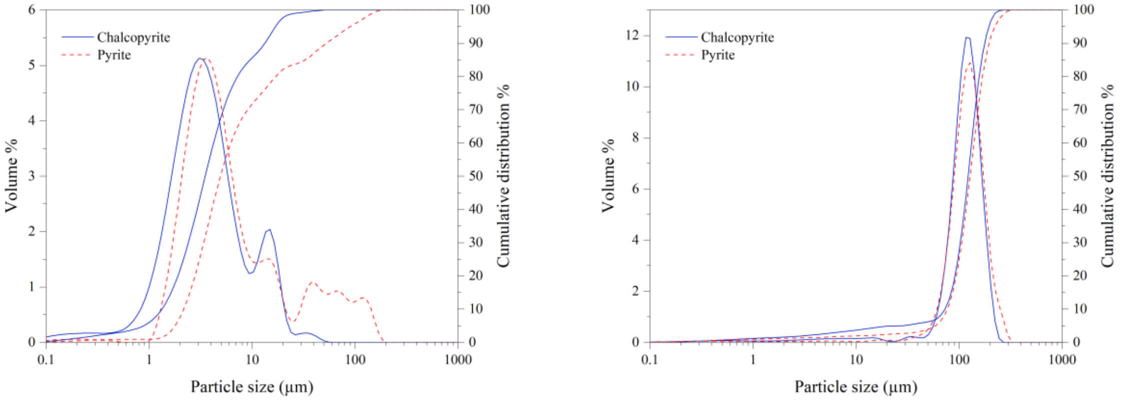

Figure S1. Particle size distribution of fine (left) and coarse (right) fractions of chalcopyrite and pyrite.

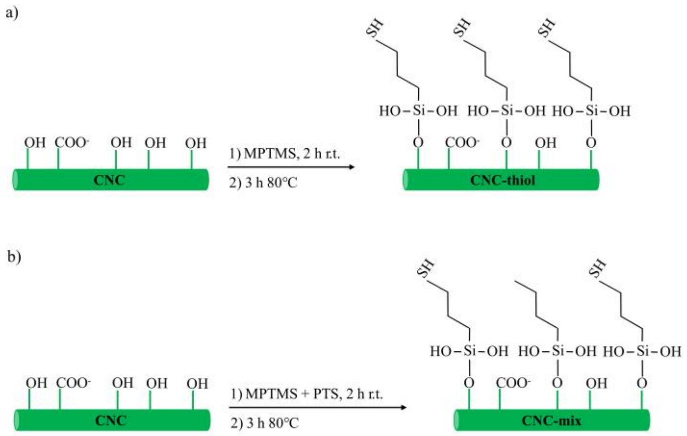

Figure S2. Synthesis of **a)** CNCs containing thiol silane group (CNC-thiol) and **b)** CNCs containing thiol and propyl silane groups (CNC-mix) via aqueous silylation.

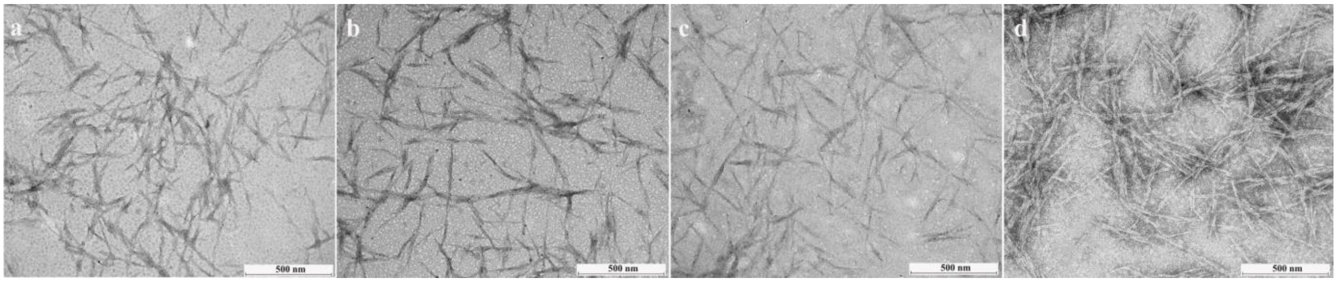

Figure S3. TEM images of functionalized CNCs: a) CNC-thiol I, b) CNC-mix I, c) CNC-thiol II, and d) CNC-mix II.

Table S2. Composition of pristine and functionalized CNCs from XPS analysis.

Sample	Surface composition (%)			
	O1s	C1s	Si2p	S2p
CNC	42.40	57.61	-	-
CNC-thiol I	43.57	54.75	0.96	0.71
CNC-mix I	43.28	52.73	2.78	1.20
CNC-thiol II	39.97	52.08	4.47	3.35
CNC-mix II	40.49	54.39	4.69	0.43

Table S3. Grafting amount and static contact angle of pristine and silylated CNCs (n = 6).

Sample	Cellulose-MPTMS (-PTS) mass ratio	Grafting amount (mmol/g)		Static contact angle (Θ)
		Si	S	
CNC	-	-	-	29.6 ± 3.6
CNC-thiol I	1:0.5	0.34	0.22	38.1 ± 3.5
CNC-thiol II	1:1	1.59	1.05	68.6 ± 1.0
CNC-mix I	1:0.5:0.5	0.99	0.37	62.2 ± 1.5
CNC-mix II	1:1	1.67	0.13	52.5 ± 3.6

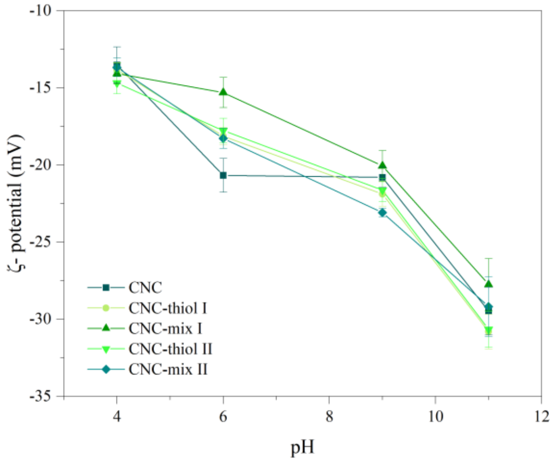

Figure S4. ζ-potential of pristine and silylated CNCs; error bars represent single standard deviation (n = 6).

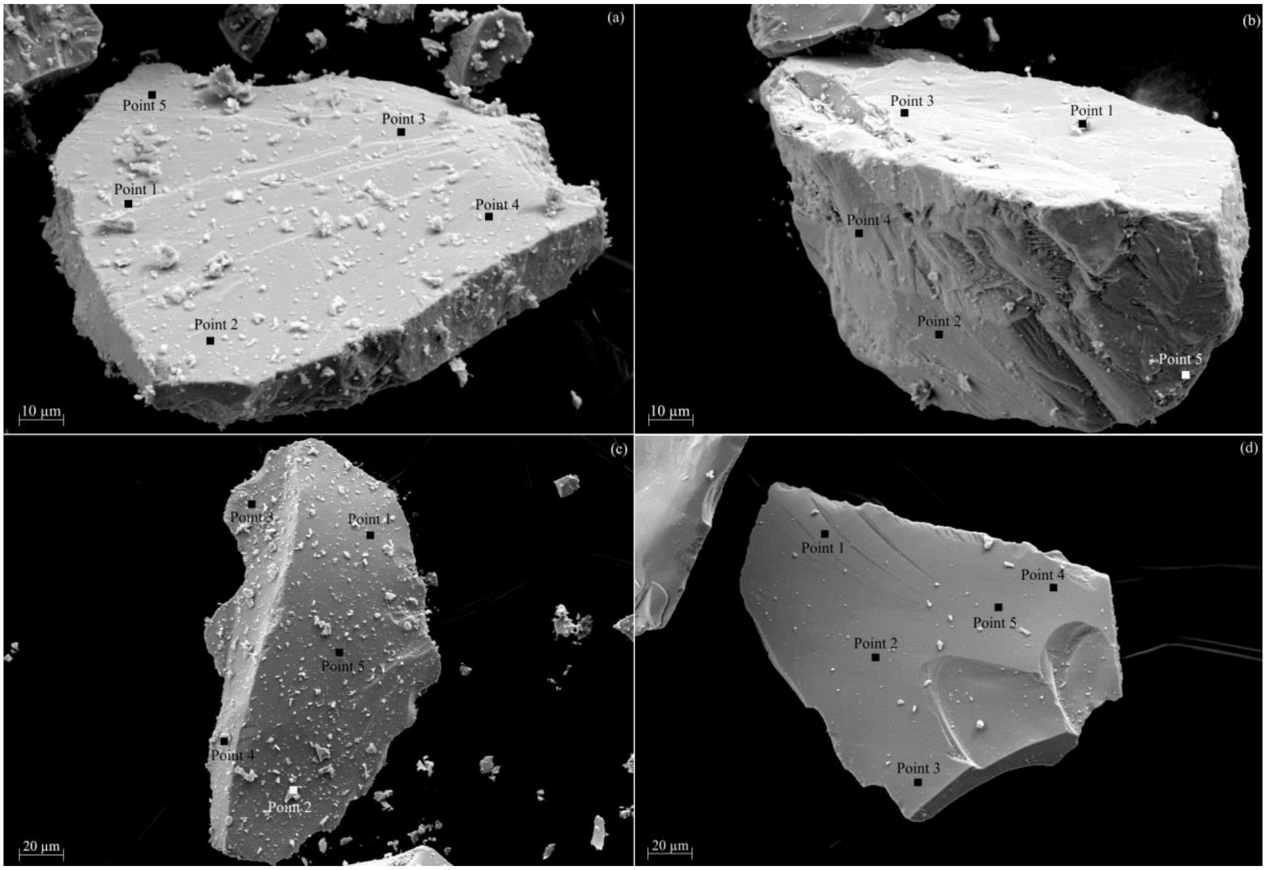

Figure S5. FESEM images of (a) chalcopyrite, (b) chalcopyrite and CNC-thiol II (2.5 mg/L), (c) pyrite, and (d) pyrite and CNC-thiol II (2.5 mg/L).

Table S4. Elemental composition (% w/w) of chalcopyrite and pyrite in the presence and absence of CNC-thiol II with standard deviations (n = 5).

	Chalcopyrite	Chalcopyrite and CNC-thiol II	Pyrite	Pyrite and CNC-thiol II
	% w/w			
O	n.d.	12.20 ± 10.34	n.d.	n.d.
Si	n.d.	0.82 ± 0.41	n.d.	n.d.
S	28.74 ± 0.15	19.12 ± 10	50.91 ± 2.79	52.51 ± 0.50
Fe	24.09 ± 0.15	36.40 ± 15.53	47.97 ± 2.59	45.87 ± 0.40
Cu	46.62 ± 0.65	31.08 ± 12.75	n.d.	n.d.
